# Neural Differentiation of Human Dental Mesenchymal Stem Cells Induced by ATRA and UDP-4: A Comparative Study

**DOI:** 10.3390/biom12020218

**Published:** 2022-01-27

**Authors:** Anastasios E. Koutsoumparis, Anastasia Patsiarika, Anastasia Tsingotjidou, Ioannis Pappas, Asterios S. Tsiftsoglou

**Affiliations:** 1 Laboratory of Pharmacology, Department of Pharmaceutical Sciences, Aristotle University of Thessaloniki, 54124 Thessaloniki, Greece; a.koutsoumparis@gmail.com (A.E.K.); panastad@gmail.com (A.P.); 2 Laboratory of Anatomy, Histology and Embryology, Faculty of Health Sciences, School of Veterinary Medicine, Aristotle University of Thessaloniki, 54124 Thessaloniki, Greece; astsing@vet.auth.gr; 3 Laboratory of Pharmacology and Toxicology, Faculty of Veterinary Science, University of Thessaly, 43100 Karditsa, Greece; ipappas@vet.uth.gr

**Keywords:** mesenchymal stem cells (MSC), stem cells from the apical papilla (SCAP), all-trans-retinoic acid (ATRA), 2-(3-ethylureido)-6-methylpyridine (UDP-4), neural trans-differentiation, immunofluorescence, recombinant human erythropoietin-alpha (rhEPO-α)

## Abstract

Human mesenchymal stem cells (MSC) are multipotent stem cells, which are isolated from various sources. Currently, there is a worldwide interest for dental MSC to be used against neurodegenerative diseases, since they derive from the neural crest and express embryonic stem cell markers. This fact prompted us to explore their potential for neural trans-differentiation in culture. We employed all-trans-retinoic acid (ATRA) and 2-(3-ethylureido)-6-methylpyridine (UDP-4) to induce neural differentiation of human MSC from the dental apical papilla (SCAP). The SCAP were exposed to either agent separately and assessed for proliferation, viability, morphology, and gene expression of the following neural-specific markers: neuron-specific enolase (*ENO2*), neurofibromin 1 (*NF1*), choline acetyltransferase (*CHAT*), tyrosine hydroxylase (*TH*), and the vesicular GABA transporter (*SLC32A1*). They were also assessed for the expression of glial fibrillary acidic protein (GFAP) and neuronal nuclear antigen (NeuN) by immunofluorescence. ATRA or UDP-4 treatment inhibited the cell growth and promoted limited cell death, but to a different extent. The addition of the neuroprotective agent recombinant human erythropoietin-alpha (rhEPO-α) enhanced the UDP-4-inducing capacity for more than three weeks. ATRA or UDP-4 treatment significantly upregulated *ENO2* and *NF1* expression, indicating neuronal differentiation. Moreover, the ATRA treatment significantly induced the upregulation of the GABAergic-specific *SLC32A1*, while the UDP-4 treatment led to the significant upregulation of the adrenergic-specific *TH*. The UDP-4 treatment induced the expression of NeuN and GFAP after four and three weeks, respectively, while the ATRA-treatment did not. Our findings indicate that SCAP can be differentiated into neural-like cells after treatment with ATRA or UDP-4 by exhibiting a disparate pattern of differentiation. Therefore, UDP-4 is suggested here as a new potent neural-differentiation-inducing compound, which, when combined with rhEPO-α, could lay the foundation for robust stem-cell-based therapies of neurodegeneration.

## 1. Introduction

Regenerative medicine has been a rapidly growing field of medicine, aiming to treat degenerative disorders with gene- and cell-based advanced-therapy medicinal products (ATMPs) [1,2]. Mesenchymal stem cells (MSC) are highly abundant multipotent stem cells derived from various tissues (Wharton’s jelly, adipose tissue, dental pulp, bone marrow, etc.), which are able to adhere onto plastic culture surfaces and differentiate into chondrocytes (cartilage forming cells), adipocytes, and osteoblasts, depending on the culture conditions and/or the tissue microenvironment [3,4,5]. MSC have attracted worldwide attention for autologous and mostly allogeneic cell-based therapies over the last years due to their unique properties, such as the following: (a) their immunomodulatory capacity and immunosuppressive potential (b) their ability to differentiate into different phenotypes, as mentioned above [5,6] (c) their capability to secrete over 40 growth factors and cytokines that contribute to tissue microenvironment and niche formation (secretome) [6].

Several studies were carried out to differentiate MSC of dental origin, as well as that of various other sources [7,8], by growing them in a culture medium with fetal calf serum (FCS), or even under serum free [9] conditions, using macromolecular factors, such as EGF, FGF, and BDNF, as neuronal cell-differentiation inducers [10,11,12]. Knowing that recombinant human erythropoietin-alpha (rhEPO-α), a well-known pleiotropic growth promoter of erythropoiesis, is also a protection factor for the survival of non-hematopoietic tissues [13,14], we were able to demonstrate that rhEPO-α promotes angiogenic/endothelial trans-differentiation of stem cells from the apical papilla (SCAP) [15]. Human SCAP originally derive from the neural crest and express the mesenchymal precursor cell markers Nestin and STRO-1, in addition to the classical MSC markers (CD44, CD73, CD90, CD117, and CD115) [6]. Thus, we investigate here whether human SCAP can be trans-differentiated into neural cells in cultures containing 15% FCS supplemented with all-trans-retinoic acid (ATRA) or UDP-4, an ureido-derivative of pyridine substituted with a 2-(3-ethyl-ureido) group at position 6 [16]. These two agents are inducers of differentiation, acting epigenetically via their own cytoplasmic receptors [17,18] and not via cell membrane-mediated signaling mechanisms, as macromolecular growth factors, such as rhEPO-α and neurotrophins, do [11,13,14]. ATRA is a well-known inducer of neuronal differentiation of embryonic stem cells, as well as MSC [19], and a promoter of synaptic plasticity of cortical neurons [20] by acting via retinoic-alpha, beta, and gamma receptors epigenetically in several tissues, including the functioning brain [21]. However, ATRA is insoluble in water and only soluble in organic solvents, where it is affected rapidly by oxidation and sunlight. UDP-4, a water-soluble molecule, has been developed in our own laboratory as a potent inducer of the erythroid differentiation of mouse erythroleukemia cells [16] and the neural differentiation of human cholinergic neuroectodermal tumor cells [22]. In the former case, UDP-4 was found to act by binding to p38 cytoplasmic proteins [17]. In this study, we compare the patterns of neural differentiation of SCAP promoted by each agent under similar culture conditions. By applying morphological criteria, assessing the levels of neuron-specific gene expression by RT–PCR, and protein expression by immunofluorescent staining with polyclonal conjugated fluorescent antibodies, we were able to demonstrate that UDP-4 is indeed a promoter of neural differentiation of SCAP, similar to ATRA, but it does so by inducing a disparate pattern of neural differentiation. Human recombinant erythropoietin-alpha (rhEPO-α) was used to maintain the survival of SCAP upon their neural differentiation in culture.

## 2. Materials and Methods

### 2.1. Cells and Culture Conditions

The human MSC used in this study (SCAP) were derived from the apical papilla of normal impacted third molars of 3 donors aged 16–18 years at the stage of root development (two thirds of the root completed). All donors were healthy, without any known diseases, were not taking any medication, were nonsmokers, and did not consume any alcohol [6]. Cells were seeded at a minimum density of 1 × 10^4^ cells/mL using alpha-modification of Eagle’s medium (α-MEM, GIBCO/Invitrogen) supplemented with 15% FBS (LONZA, Basel, Switzerland), 100 mM L-ascorbic acid phosphate, 2 mM L-glutamine, 100 IU/mL penicillin, 100 mg/mL streptomycin, and were incubated at 37 °C in 5% CO_2_. Cultured SCAP, in passage 2–4, were used for all experiments.

### 2.2. Compounds

**Figure 1:**
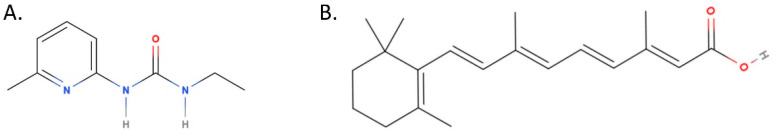
Molecular structures of (**A**) UDP-4 and (**B**) ATRA. UDP-4 along with many other ureido-derivatives of pyridine bears hydrophilic functional groups that ensure water solubility.

UDP-4 (2-(3-ethylureido)-6-methylpyridine) (Figure 1A) was synthesized in our laboratory of Pharmacology (School of Pharmacy, AUTh) by Dr. Ioannis Pappas [16] and was diluted in a mildly acidic (pH = 5.5) aqueous solution of hydrochloric acid. All-trans-retinoic acid (ATRA) (Figure 1B); (Retinoic acid ≥ 98% (HPLC) powder, Sigma-Aldrich) was diluted in 100% ethanol and stored at 4 °C, protected from direct sunlight. Recombinant human erythropoietin-alpha (Eprex^®^, inj. sol. 10,000 IU/mL, Jansen-Cilag, Beerse, Belgium) was stored at 4 °C.

### 2.3. Evaluation of Gene Expression with RT–PCR

Untreated SCAP and cells treated with ATRA or UDP-4, were assessed by RT–PCR for the expression of genes considered as markers of neuronal differentiation. These include the following: choline acetyl-transferase (*CHAT*) [22], tyrosine hydroxylase (*TH*), vesicular GABA transporter (*SLC32A1*), neuron-specific enolase (*ENO2*) [23], and neurofibromin 1 (*NF1*) [24]. *GADPH* was used as the internal control. Total RNA was extracted with the guanidinium thiocyanate–phenol–chloroform method from an adequate number of cultured cells. RNA was then treated with DNase I, assessed for quality (agarose electrophoresis) and quantity (Nano-drop 200, Thermo Fisher Scientific, Waltham, MA, USA), and was used as a template for cDNA synthesis and subsequently RT–PCR (One Taq^®^ RT–PCR kit, BioLabs Inc., Ipswich. MA, USA) under the following conditions: (1) 95 °C for 30 s, (2) 94 °C for 20 s, (3) T_a_ (varies) for 30 s, (4) 68 °C for 25 s, (5) back to step 2 for 35 times, and (6) 68 °C for 5 min. Primers were designed to span exon–exon junctions and avoid hairpin formation using the NCBI gene database, along with Primer-BLAST and OligoCalc bioinformatics tools, and were added at a final concentration of 5 μM. Primer sequences were as follows:

*ENO2* (NM_001975.2)–Sense: TGTGCACAGGCCAGATCAAG
// Antisense: GTGCGGAACCCCAATGAGTA and T_a_ = 60 °C
*NF1* (NM_001042492.2)–Sense: AACGAGTGTCTCATGGGCAG
// Antisense: GTGCAGAGGCGAGTCCTTAT and T_a_ = 60 °C
*GAPDH* (NM_002046.5)–Sense: GAAGGTGAAGGTCGGAGT
// Antisense: GAAGATGGTGATGGGATTTC and T_a_ = 56 °C
*TH* (NM_000360.3)–Sense: CCAAGTTCGACCCTGACCTG
// Antisense: CAATGTCCTGCGAGAACTGC and T_a_ = 58 °C
*CHAT* (NM_020549.4)–Sense: TGGGCTCTTCTCCTCCTACC
// Antisense: CCGGTTGGTGGAGTCTTTCA and T_a_ = 58 °C
*SLC32A1* (NM_080552.2)–Sense: GCCATCCAGGGCATGTTCG
// Antisense: AGCTCGATGATCTGCGCTAC and T_a_ = 58 °C

After agarose electrophoresis, we received a single product band at the predicted molecular weight in each positive sample. Densitometry of various genes was performed with the ImageJ 1.49 (NIH) software.

### 2.4. Immunocytochemistry

SCAP treated with ATRA or UDP-4, were immunostained for glial fibrillary acidic protein (GFAP) and neuronal nuclear antigen (NeuN) using monoclonal fluorescent-conjugated antibodies, as follows: Cells attached on glass coverslips (DIOLAB, Athens, Greece) were fixed with 4% PFA for 20 min and then permeabilized using 0–1% of Triton-X 100 in PBS. Next, blocking solution containing 10% NGS and 0.05% Triton-X 100 in PBS was applied on the coverslips for 1 h. Cells were incubated with anti-GFAP (1:200) (rabbit polyclonal, 1:400; Dako: #Z03334; RRID: AB-100113382) and anti-NeuN (1:500) (rabbit polyclonal 1:1000. Millipore; #AN78; RRID; AB-10807945) for 1 h, diluted in 1% NGS in PBS. Cells were then washed with 1% NFS in PBS and incubated for 1 h at RT with the appropriate secondary antibodies (goat anti-mouse 488 Biotinum; #20010; RRID: AB-10844384) and goat anti-rabbit 555 (Biotinum; #20033; RRID, AB 10599671) diluted at 1:500 in 1% NGS in PBS. Positive and negative controls were used. Finally, mounting medium containing DAPI was used for the immunostaining of the nuclei. Cells stained red for GFAP and green for NeuN were visualized and examined under a fluorescent microscope. Information for the antibody characterization was derived from the published work of Bekiari et al. (2020) [25]. GFAP is an intracytoplasmic filamentous protein of astrocytes, proven to be a specific marker of cells of astrocyte origin, as the radial glia-like neural stem cells of the adult brain [26]. The polyclonal anti-GFAP (glial fibrillary acid protein) antibody (Dako; #Z0334) RRID: AB 100113382) was provided as purified immunoglobulin fraction of rabbit antiserum and the immunogen used was GFAP isolated from cow spinal cord. It was solid-phase absorbed with human and cow serum proteins and in crossed immunoelectrophoresis showed one distinct precipitate (GFAP) with cow brain extract (DAKO antibody data sheet). NeuN protein shows a strong cytoplasmic and nuclear concentration and is the most widely used marker of vertebrate central nervous system (CNS) and the peripheral (PNS) neuronal cells, as described by Rao et al. (2005) [27]. The polyclonal anti-NeuN (Neuronal Nuclei; Millipore; #ABN78; RRDI: AB 10807945) antibody was raised in rabbits after immunization with GST-tagged recombinant mouse NeuN protein and was evaluated by western blotting in mouse brain nuclear extract (Millipore antibody datasheet). It recognized the DNA-binding NeuN protein at the N-terminus. The goat anti-mouse 488 (Biotinum; #20010; RRId: AB 10854383) and the goat anti rabbit 555 (Biotinum; #20033; RRID: AB 10559671) were labeled with fluorescent agent and used as the secondary antibodies.

### 2.5. Statistical Analysis

Phenotypes were assessed for statistical significance using the independent-sample *t*-test in OriginPro. The resulting *p*-values were corrected for multiple comparisons using the Bonferroni method at the significance level α = 0.05. Wherever applicable, ***: *p* < 0.001, **: *p* < 0.01, * *p* < 0.05, n.s.: *p* > 0.05, and error bars correspond to the standard error of the mean.

## 3. Results

### 3.1. Treatment of SCAP with ATRA or UDP-4 Affects Cell Growth and Viability

**Figure 2:**
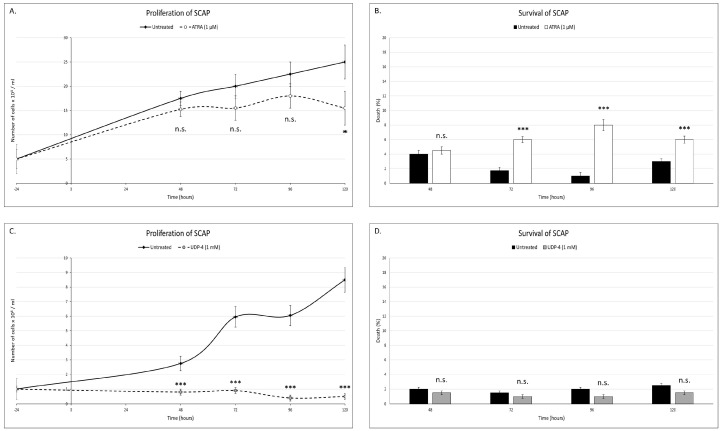
ATRA (1 μM) affects the (**A**) proliferation and (**B**) survival of SCAP. Seeding density was 5 × 10^4^ cells/mL and ATRA was added at 0 h. Treatment with ATRA increased the doubling time of the culture by 32 h and significantly increased cell death. UDP-4 (1 mM) affects the (**C**) proliferation and (**D**) survival of SCAP. Seeding density was 1 × 10^4^ cells/mL and UDP-4 was added at 0 h. Treatment with UDP-4 halts the proliferation of the cell culture but did not affect cell death. ***: *p* < 0.001, * *p* < 0.05, n.s.: *p* > 0.05, and error bars correspond to the standard error of the mean.

In the first series of experiments, the optimal concentration of ATRA and UDP-4 was investigated. This is the lowest concentration that induces morphological differentiation and allows for the highest cell viability. It was found to be 1 μM for ATRA and 1 mM for UDP-4, respectively. The treatment with 1 μM of ATRA increased the cell culture doubling time by 32 h (from 52 h in the untreated SCAP to 84 h in the ATRA-treated SCAP), suggesting that ATRA inhibits the cell-growth rate. This resulted in a significantly reduced cell population after 120 h of treatment (Figure 2A). Also, the ATRA treatment led to significantly more cell death, up to an 8-fold increase at 96 h (Figure 2B). The treatment with 1 mM of UDP-4 forces the culture in a steady state of population equal to the seeding density (Figure 2C), suggesting that UDP-4 blocks cellular proliferation very fast. With regard to the viability, the UDP-4 treatment did not show any significant difference from the untreated SCAP, indicating that UDP-4 is comparatively less toxic to the cells than ATRA (Figure 2D). It should be noted here that, at all of the time points, the culture confluence was ≤80%, so that contact inhibition phenomena were of no significant importance. All measurements were conducted in three biological replicates.

### 3.2. ATRA or UDP-4 Treatment Changes Cellular Morphology into Neural Phenotypes

**Figure 3:**
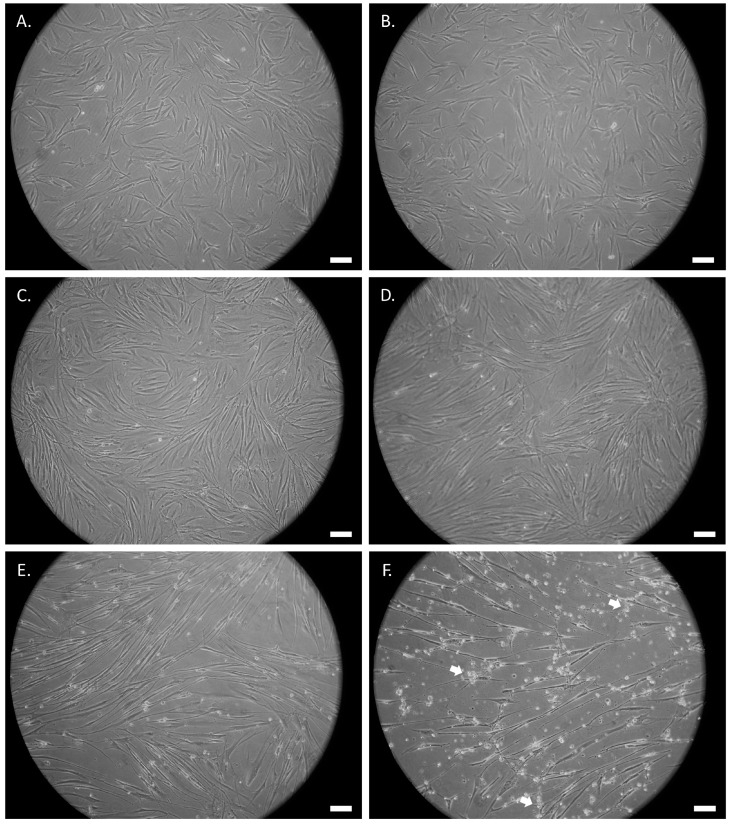
Phase contrast microscopic images of SCAP treated with 1 mM of UDP-4 cultured for (**A**) 0 h (untreated), (**B**) 72 h, (**C**) 96 h, (**D**) 7 days, (**E**) 14 days, and (**F**) 21 days showing the progressive development of elongated neural-like morphology. White arrows denote cellular stress. Scale bars correspond to 50 µm.

**Figure 4:**
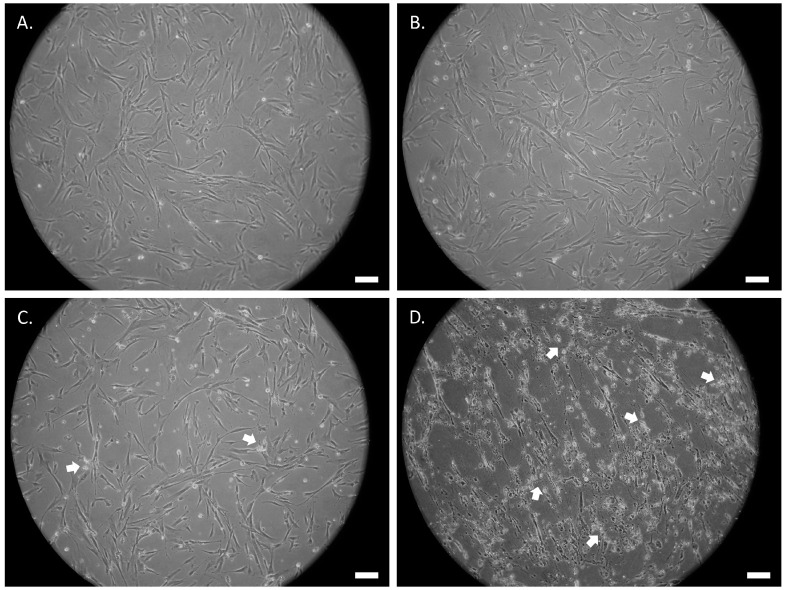
Phase contrast microscopic images of SCAP treated with 1 μM of ATRA cultured for (**A**) 0 h (untreated), (**B**) 72 h, (**C**) 96 h, and (**D**) 7 days showing the progressive development of elongated neural-like morphology. White arrows denote cellular stress. Scale bars correspond to 50 µm.

The morphological changes of the untreated and the ATRA- or UDP-4-treated SCAP were assessed by examination under an optical microscope at 20× magnification. Following the treatment with either compound, the cells start to deviate from the standard fibroblast-like MSC morphology and adopt an elongated, thinner form, which resembles that of axons and dendrites of neuron-like cells (Figure 3 and Figure 4). This process is accompanied with stress that manifests as circular aggregates in the cell culture. Stress is evident in the ATRA-treated cells, even at 72 h of treatment (Figure 4B), and becomes severe after seven days (Figure 4D), while in the UDP-4-treated cells there is less stress, which only becomes apparent after 14 days of treatment (Figure 3E) and peaks at 21 days (Figure 3F). Overall, UDP-4 induces neuron-like phenotypes in a “healthier” way than ATRA.

### 3.3. UDP-4 Treatment Increases Neuronal-Specific Gene Expression of ENO2 and NF1

**Figure 5:**
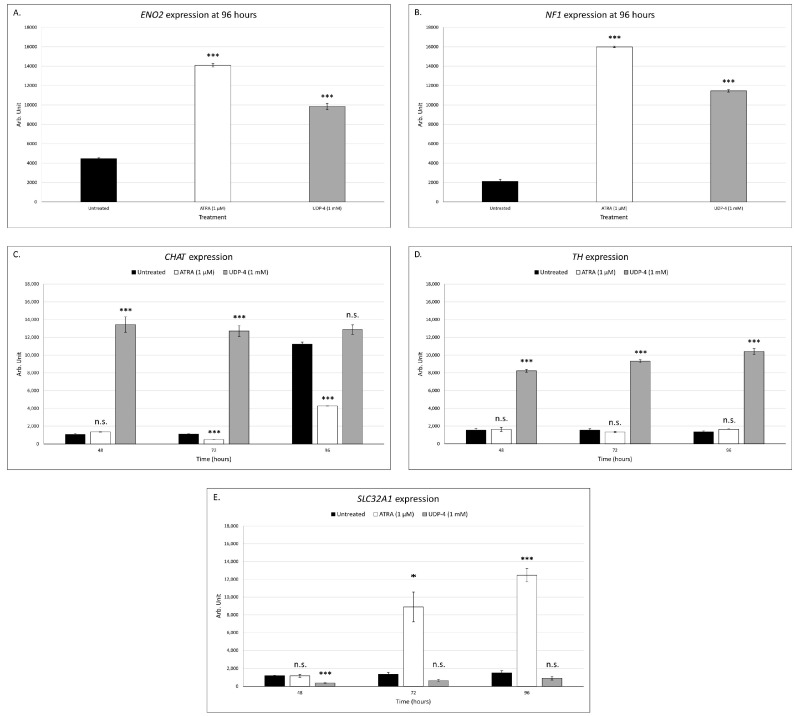
RT–PCR assessment of neuronal gene expression of SCAP treated with ATRA (1 μM) or UDP-4 (1 mΜ). (**A**) *ENO2* expression was significantly upregulated after 96 h of treatment with ATRA or UDP-4. (**B**) *NF1* expression was significantly upregulated after 96 h of treatment with ATRA or UDP-4. (**C**) *CHAT* expression was significantly downregulated over the course of treatment with ATRA, while it was significantly upregulated as early as 48 h after treatment with UDP-4. (**D**) *TH* expression was not altered by treatment with ATRA, while it was significantly upregulated over the course of treatment with UDP-4. (**E**) *SLC32A1* expression was significantly upregulated over the course of treatment with ATRA, while it was not affected by the UDP-4. ***: *p* < 0.001, * *p* < 0.05, n.s.: *p* > 0.05, and error bars correspond to the standard error of the mean.

In order to provide further evidence that UDP-4 induces neuronal differentiation, we performed RT–PCR analysis to assess the expression pattern of two genes, specific for neuronal cells. *ENO2* encodes for neuron-specific enolase, which is expressed selectively in mature neurons and cells of neuronal origin [8]. *NF1* encodes for neurofibromin 1, which is expressed in oligodendrocytes and Schwann cells [9]. Untreated SCAP express *ENO2* and *NF-1* at a basal level. A significant increase in the expression of both of these markers was reported, following the treatment with ATRA or UDP-4 (Figure 5A,B). These findings indicate that ATRA and UDP-4 promote neuronal differentiation. Moreover, in order to determine the specific kind of differentiated neurons, we performed RT–PCR analysis for choline acetyltransferase (*CHAT*), tyrosine hydroxylase (*TH*), and the GABA vesicular transporter (*SLC32A1*), which serve as cholinergic, adrenergic, and GABAergic neuronal differentiation markers, respectively. We can conclude that the UDP-4 treatment is likely to promote differentiation into adrenergic neurons because there is a significant increase in *TH* levels (Figure 5D), while the ATRA treatment is likely to promote GABAergic differentiation because there is a significant increase in *SLC32A1* levels (Figure 5E).

### 3.4. Erythropoietin Increases Survival of UDP-4 Treated Cells

Erythropoietin is a hematopoietic growth factor of pleiotropic functions and its role as a neuroprotective/survival factor has been well established [13,14,15]. Thus, we utilized rhEPO-α at 40 IU/mL to prolong the lifespan and reduce the stress of our UDP-4 induced neuronal-like cells. The cells were cultured for seven days with UDP-4 (1 mM). At that point, rhEPO-α was added at 40 IU/mL and cells were further cultured for 14 additional days.

**Figure 6:**
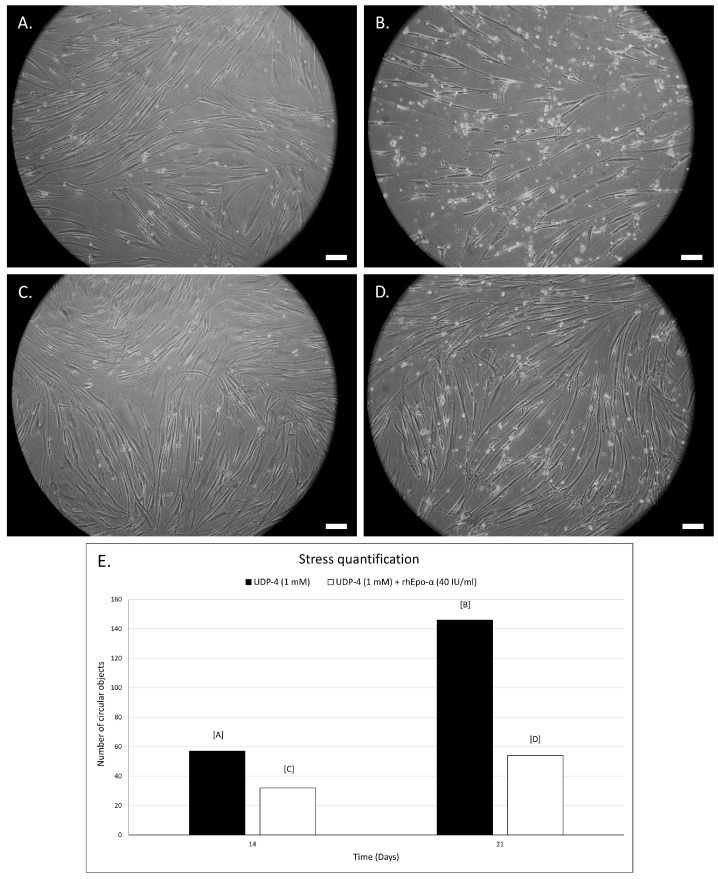
Phase contrast microscopic images of SCAP treated with 1 mM of UDP-4 cultured for (**A**) 14 days and (**B**) 21 days showing decreased population and signs of cell stress. However, after washing-out UDP-4 after 7 days and treating cells with 40 IU/mL rhEPO-α for an additional (**C**) 7 days and (**D**) 14 days, cell density increases, whereas cell-stress signs decrease. (**E**) Quantification of cellular stress, by automatic detection of circular objects with ImageJ, shows strongly reduced stress after rhEPO-α treatment. Scale bars correspond to 50 µm.

The growth medium was not renewed at any point. After 14 total days of culture, cell stress was evident both in the untreated and the rhEPO-α-treated samples (Figure 6A,C). However, the cell density was clearly higher in the rhEPO-α treated samples after 21 days (Figure 6B,D) and the cellular aggregates were greatly reduced (Figure 6E). Also, the rhEPO-α treatment resulted in a viable and robust culture even after 30 days (end of the experiment), while lack of rhEPO-α led to a UDP-4 stress induced cell death shortly after the 21 day mark (data not shown). This was not the case with the ATRA-treated cells, as their survival was problematic due to stress, even in the presence of rhEPO-α. These findings indicate that rhEPO-α acts protectively, extending the survival and the welfare of the UDP-4 induced neuronal-like cells.

### 3.5. UDP-4 Treatment Increases the Neuronal-Specific Expression of GFAP and NeuN

**Figure 7:**
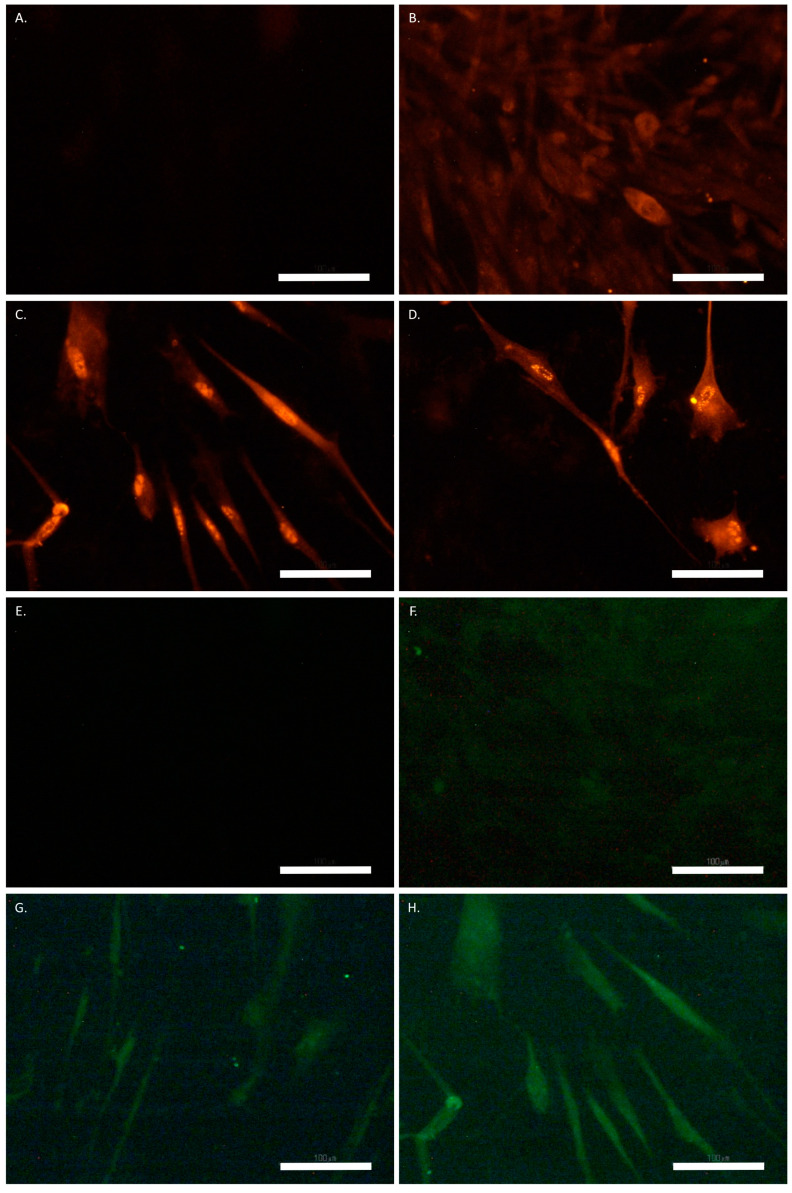
SCAP treated with UDP-4 and stained for GFAP (red) and NeuN (green). (**A**) Untreated cells stained with the secondary antibody for GFAP-isotype control. (**B**) Untreated cells stained for GFAP, showing basal expression. (**C**) UDP-4-treated cells after 3 weeks, showing medium expression of GFAP. (**D**) UDP-4-treated cells after 4 weeks, showing strong expression of GFAP. (**E**) Untreated cells stained with the secondary antibody for NeuN-isotype control. (**F**) Untreated cells stained for NeuN, showing no expression. (**G**) UDP-4-treated cells after 2 weeks, showing basal expression of NeuN. (**H**) UDP-4-treated cells after 3 weeks, showing medium NeuN expression. Experiments were conducted in duplicates. Scale bars correspond to 100 µm.

The immunostaining for GFAP showed basal expression, while there was no signal detected for NeuN in the untreated SCAP (Figure 7B,F). Also, no labelling was present in the untreated cells stained with the secondary antibody for GFAP or NeuN as an alone-isotype control (Figure 7A,E). The basal expression of GFAP was present in both the ATRA- and the UDP-4-treated cells after one and two weeks, which increased at three weeks to medium levels (Figure 7C). After four weeks of UDP-4 treatment, we observed a strong expression of GFAP (Figure 7D), while the ATRA treatment resulted in no detectable levels. With regard to NeuN, no signal was detected after one week with either treatment. The basal expression of NeuN was observed after two weeks of UDP-4 treatment (Figure 7G), while the ATRA-treated cells still produced no detectable signal. Finally, after three and four weeks of treatment with UDP-4, there was medium expression of NeuN (Figure 7H), while there was still no expression in the ATRA-treated cells. The above results are summarized in Table 1.

## 4. Discussion

Early studies suggested that bone marrow cells can be intraconverted to different cell types, including neurons [28]. This proposed intraconversion of cell phenotypes attracted worldwide attention and promoted further stem cell research leading to the development of regenerative medicine [1] and gene- and cell-based advanced cell products aimed for therapy of degenerative and genetic disorders [2]. Human and animal mesenchymal multipotent stem cells (MSC) have been at the center of interest as tools for tissue regeneration [3,4]. Moreover, the fact that dental MSC are derived from the neural crest and express Nestin and embryonic cell markers, such as STRO-1 [29], has made them able to facilitate tissue regeneration and repair in the CNS and PNS [7,9,10,11]. Indeed, now evidence exists that dental MSC grown in vitro with growth factors, such as FGF2 and BDNF, are able to give birth to functional neurons [11,12,30]. Studies with ATRA indicated that in some cases, this small-molecular-weight compound promotes neural differentiation [31].

Taking all of these observations into account, we asked whether other small-molecular-weight compounds that act epigenetically may also promote neuronal differentiation, perhaps with even higher potency. As presented in this comparative study, we investigated the potential of UDP-4 to induce neural differentiation in human SCAP. ATRA, was utilized as a positive control, due to its ability to stimulate neural differentiation [31]. Our results provide evidence that UDP-4, a novel inducer of differentiation, was shown to be superior in this case because of its chemical characteristics. It is easy to synthesize and dissolve in aquatic solutions. It is not affected by oxidation and electromagnetic radiation, as we have demonstrated [17]. It is far less toxic for MSC than ATRA, even at a 1000-fold higher concentration. Although treatment with each compound resulted in lower cell growth rate (Figure 2A,C), the ATRA-treated cultures were short-lived and showed considerably higher cell stress compared to the UDP-4-treated cultures (Figure 2C,D, Figure 3 and Figure 4). Characteristic, neuron-like, elongated morphology became apparent in the UDP-4-treated cells after seven days (Figure 3D). RT–PCR analysis confirmed that the mRNA expression of the neural-specific marker genes was upregulated (*ENO2*, *NF1*), starting at 96 h (Figure 5A,B). The further search for neuron-specific differentiation revealed that UDP-4-treatment promotes adrenergic-like neuron formation by upregulating *TH* (Figure 5D), whereas ATRA treatment promotes GABAergic-like neuron formation, characterized by the upregulation of *SLC32A1* (Figure 5E). The cholinergic-specific marker *CHAT* was also upregulated after UDP-4 treatment as early as 48 h, however, it did not exceed the levels of the untreated samples after 96 h (Figure 5C). This measurement also revealed that *CHAT* is constitutively upregulated in untreated SCAP over time. Since there were no other morphological or molecular signs of neural differentiation in the untreated SCAP and the partial upregulation of *CHAT* took place with the ATRA treatment as well (Figure 5C), we consider this a normal aspect of SCAP biology. Perhaps SCAP start producing acetylcholine as part of their secretome during the aging process. Therefore, we could theorize here that either UDP-4 not only drives the differentiation process towards the adrenergic neuron fate, but also does so in an accelerated manner (with *CHAT* expression being a “maturation” marker here) or UDP-4 drives the differentiation towards a sympathetic neuron precursor that has the potential to differentiate further into one of two fates, cholinergic or adrenergic. Both of the suggestions made above could hold together as well. Moreover, we tested the potential of rhEPO-α as a neuroprotective agent based on the existing literature [13,14], in order to protect the UDP-4-treated cells from the differentiation- and/or aging-induced stress. Remarkably, we showed that the addition of rhEPO-α maintained the neural-like phenotype that was induced from UDP-4 and rejuvenated the stressed cells (Figure 6). Finally, after immunostaining, we observed that cells treated with ATRA expressed basal levels of GFAP, similar to other types of MSC of dental origin (e.g., DPSC) [12], while NeuN was hardly detectable (Table 1). Although we found that ATRA treatment upregulated the expression of genes related to the neural phenotype, such as *ENO2* and *NF1*, as well as the GABAergic-specific *SLC32A1*, its inability to induce the expression of NeuN suggests the molecule’s poor ability to differentiate dental MSC into neurons. We were also able to show that, after treatment with UDP-4, both the expression of GFAP and NeuN is upregulated (Figure 7; Table 1), strongly indicating the neural trans-differentiation of SCAP. GFAP, except for astrocytes, is also enriched in radial glia [25,26,27], while NeuN constitutes a characteristic pan-neuronal marker [32]. Radial glia are key progenitor cells in the nervous system. They can divide asymmetrically to produce a radial glial cell and a differentiated neuron [33]. With this in mind, we propose here that following UDP-4 treatment, MSC are differentiated towards radial glial cells with the potential to commit in the sympathetic-neuron precursor fate. In any case, GFAP-positive reactivity has already been detected on neurons (NeuN expression; Table 1), which is consistent with previous studies [25]. Additionally, Nestin-positive neural precursors can give rise both to astrocytes and neurons, hence, the differentiation of SCAP is consistent with these studies [34].

## 5. Conclusions

UDP-4 appears to be a highly promising agent to facilitate differentiation of MSC into neuronal cells. The molecule’s favorable characteristics include the following: water solubility, ease of synthesis and large-scale production, and low cytotoxicity (particularly when combined with the cytoprotective rhEPO-α). UDP-4 is, overall, more potent than the already established ATRA in inducing neural differentiation. These properties support the suitability of UDP-4 as a pharmaceutical agent (alone or in combination with selective growth factors) for cell-based therapies and the repair of neuron degeneration [35].

## Figures and Tables

**Table 1:** Expression levels of GFAP and NeuN in SCAP after treatment with UDP-4 or ATRA.

		GFAP	NeuN
Untreated		+	+/−
UDP-4	1 week	+	+/−
	2 weeks	+	+
	3 weeks	++	++
	4 weeks	+++	++
ATRA	1 week	+	+/−
	2 weeks	+	+/−
	3 weeks	++	+/−
	4 weeks	+/−	+/−

+/−: no signal; +: basal expression; ++: medium expression; +++: strong expression.

## Data Availability

Not applicable.
